# A viroid-like RNA can be transmitted among different *Trichoderma* species affecting their antagonistic capacity

**DOI:** 10.1128/mbio.00221-26

**Published:** 2026-06-15

**Authors:** Cristina Formiglia, Marco Forgia, Beatriz Navarro, Francesco Di Serio, Nadia Serale, Safa Oufensou, Virgilio Balmas, Quirico Migheli, Niccolò Miotti, Olga Rueda, Federica Bono, Marcos de la Peña, Massimo Turina

**Affiliations:** 1 Institute for Sustainable Plant Protection, National Research Council of Italy, Torino, Italy; 2 Department of Life Sciences and Systems Biology, University of Turin 9314, Turin, Italy; 3 Institute for Sustainable Plant Protection, National Research Council of Italy 111213 , Bari, Italy; 4 Department of Agricultural Sciences and NRD—Desertification Research Center, University of Sassari 9312, Sassari, Italy; 5 Department of Molecular and Translational Medicine, University of Brescia 9297, Brescia, Italy; 6 Instituto de Biología Molecular y Celular de Plantas, Universidad Politécnica de Valencia-CSIC 54403, Valencia, Spain; 7 Department of Plant Protection, School of Agriculture, The University of Jordan 54658, Amman, Jordan; Okayama University 53357, Kurashiki, Okayama, Japan

**Keywords:** viroids, fungi, horizontal transmissions, antagonism, RNA silencing, *Trichoderma*

## Abstract

Viroids are small, circular non-coding RNAs that autonomously replicate in plants, exploiting host cellular machinery for replication and spread. Recent studies reveal that viroid-like agents can infect filamentous fungi, suggesting cross-kingdom interactions. In this study, we report the discovery and the characterization of TsvlRNA1 in *Trichoderma spirale*. TsvlRNA1 is a 712-nt-long viroid-like RNA containing a hammerhead ribozyme in one polarity strand. Bioinformatic data, molecular validation, and reverse genetics experiments demonstrate that TsvlRNA1 is circular with an active ribozyme essential for replication. TsvlRNA1 replicates autonomously and transmits between *Trichoderma* species, eliciting 20–22 nt viroid-derived small RNAs consistent with RNA silencing targeting. The biocontrol capacity of *Trichoderma* against *Rhizoctonia solani* is variably modulated by TsvlRNA1, with effects ranging from positive to negative depending on host strain. In *T. spirale*, data suggest that TsvlRNA1 negatively affects antagonistic potential. TsvlRNA1 transmission via horizontal routes is prevalent, and the viroid-like RNA fails to infect plant hosts experimentally. These results highlight the as-yet underappreciated ecological and functional diversity of viroid-like agents in fungi, with implications for fungal biology, biocontrol, and genotype-phenotype relationships in eukaryotes.

**IMPORTANCE:**

Species of the fungal genus *Trichoderma* play a central role in sustainable agriculture by controlling fungal plant pathogens and supporting plant growth. For this reason, *Trichoderma*-based products represent a substantial share of the global market for microbial biofungicides. Viroids are the smallest known infectious agents, and their presence in filamentous fungi has only recently been discovered. Consequently, little is known about their biology, transmission, or interactions with fungal hosts. In this study, we describe TsvlRNA1, a viroid-like RNA associated with *T. spirale*, representing only the second viroid-like RNA to be biologically characterized in fungi. We show that TsvlRNA1 can influence the ability of *Trichoderma* to inhibit *Rhizoctonia solani*, a major plant pathogen, demonstrating its biological relevance. Unexpectedly, TsvlRNA1 can be transmitted between different *Trichoderma* species. This finding raises concerns about the possible transfer of genetic traits between fungi, including potentially those related to fungicide resistance, with important implications for agricultural biocontrol.

## INTRODUCTION

Viroids, first identified as plant pathogens in the early 1970s (1), are recognized as the smallest replicators. They consist of a covalently closed circular (ccc) RNA ranging from 234 to 506 nucleotides in length, exhibiting high self-complementarity, thus assuming a rod- or branch-shaped conformation (2–4). Viroids lack protein-coding capacity, and some of them contain a self-cleaving ribozyme in each polarity strand that catalyzes a site-specific cleavage of viroid oligomeric RNAs during replication (5–8).

Viroids are classified into two families (6, 8): *Pospiviroidae*, which groups viroids replicating and accumulating within the nucleus, and *Avsunviroidae*, which includes viroids that replicate and accumulate in chloroplasts and possess hammerhead ribozymes (HHrbz) (9, 10).

Replication occurs through a rolling-circle replication (RCR) mechanism comprising three steps: RNA transcription, processing, and ligation. *Pospiviroidae* follow an asymmetric RCR pathway, catalyzed by host enzymes such as RNA polymerase II, RNase III-like, and DNA ligase I (2, 8). In contrast, *Avsunviroidae* follow a symmetric RCR pathway mediated by the nuclear-encoded and plastid localized RNA polymerase, self-cleaving ribozymes, and the chloroplast tRNA ligase (11, 12). While in the symmetric RCR pathway, both genomic and antigenomic RNAs are cleaved and circularized, in the asymmetric pathway only genomic RNAs undergo these steps (2, 13, 14).

Viroid-like RNA (vdlRNA) elements include plant virus satellite RNAs, retroviroids, retrozymes, and members of the realm *Ribozyviria* with larger genomes and protein-coding capacity (15–19). An example is the hepatitis delta virus, a human pathogen that encodes the delta antigen (20). Recently, a diverse array of viroid-like agents, including ambiviruses, some mitoviruses, Zeta viruses, and Obelisks, has been characterized, extending the host range of vdlRNAs to fungi and bacteria (5, 21–23).

The fungal genus *Trichoderma* (teleomorph *Hypocrea*, Hypocreales, Ascomycota) is widely distributed across ecosystems, including agricultural fields and decaying plant material (24). Nearly 500 species have been identified globally (25), many of which are exploited for biocontrol of fungal phytopathogens through mechanisms like competition, antibiosis, mycoparasitism, induction of plant defense responses, and promotion of plant growth (26). Additionally, *Trichoderma* species are utilized in the production of antibiotics, enzymes, and biofuel (27, 28) and in the bioremediation of xenobiotic compounds in water and soil (29).

Fungal cytoplasmic infectious elements (mycoviruses and vdlRNAs) are transmitted horizontally within populations via hyphal fusion. A barrier to hyphal fusion is the vegetative incompatibility (VIC), which regulates self/non-self recognition through *het/vic* genes and limits horizontal mycovirus transmission (30). Compatibility among *Trichoderma* isolates has been examined via heterokaryon—structures in which genetically distinct nuclei coexist within a common cytoplasm—formation through hyphal anastomosis or protoplast fusion (31, 32). However, most studies precede molecular techniques, which expanded the genus to 478 species (33). Recent analyses of vegetatively compatible groups (VCG) in *Trichoderma* are scarce and largely based on morphological observations (34).

This study investigates the presence of a vdlRNA sequence in a previously characterized collection of *Trichoderma* spp. isolates from Sardinian uncultivated soils (35, 36). We characterized a vdlRNA isolated from *T. spirale*, previously detected as ORFan segment of putative viral nature (35) and now named Trichoderma spirale viroid-like RNA 1 (TsvlRNA1). An infectious clone was successfully developed, enabling demonstration of its autonomous replication and horizontal transmission to different *Trichoderma* species following co-culture transfection. This achievement allows for the first time to prove the role of a vdlRNA in influencing the antagonistic properties of *Trichoderma* spp.

## MATERIALS AND METHODS

### Origin of fungal isolates, ORFan/ribozyme analysis of the sequence read archive, and *in vitro* culture on potato dextrose agar

The fungal isolates used in this study, belonging to the genus *Trichoderma*, are part of a collection housed at the University of Sassari, Italy, and assembled since 2009 (36). An ORFan identified in the study by Pagnoni et al. (35) was further investigated using the INFERNAL algorithm on Serratus cloud computing platform (5), designed for the detection of circular molecules and ribozymes. The strain *T. spirale* (T45) found to carry the vdlRNA was subjected to Next-Generation Sequencing analysis (Bioproject PRJNA1303385, Accession Number SRR34918256).

A viroid-free isogenic *T. spirale* (T45neg) colony was obtained by preparing a conidial suspension from TsvlRNA1-positive T45 and selecting physically isolated colonies, which were screened for the absence of vdlRNA.

Isolates used in this study were placed on Potato Dextrose Agar (PDA; Sigma-Aldrich, St. Louis, MO, USA) medium and incubated at 28°C for 8–10 days under near-ultraviolet light to induce conidiation.

### Identification of different *Trichoderma* species

Sequence data from the internal transcribed spacer (ITS) regions, the RNA polymerase II subunit (RPB2), and the translation elongation factor 1-α gene (TEF1-α) were used to perform a multi-locus molecular phylogenetic analysis, thus identifying the *Trichoderma* species.

Genomic DNA from each strain was extracted from fresh mycelium, following the thermolysis method described by Zhang et al. (37).

PCR amplification of the ITS, RPB2, and TEF1-α gene fragments was performed using OneTaq DNA polymerase (NEB, USA) and gene-specific primers (Table S1). Amplicons were purified with the “Zymo gel DNA recovery kit” (Zymo research, Irvine, CA, USA) and bidirectionally sequenced by Biofab Research (Rome, Italy).

For species identification, ITS, RPB2, and TEF1-α sequences from isolates T22, T36, T45, T71, TO71B, T84, T99, and T100 were aligned with reference sequences (Table S2). MUSCLE alignment was performed in MEGA11, and a maximum-likelihood phylogenetic tree was generated with IQ-TREE (38), with clade support assessed by 1,000 bootstrap replicates using *Protocrea pallida* as the outgroup.

### Transformation vector assembly

Inverse PCR with primers CHV1-25Rev and CHV1-12700-For (Table S1) released the CHV1 genome from the transformation plasmid pHX9 (39), generating pHX9-nv with multiple cloning sites under the *Cryphonectria parasitica gpd-1* promoter/terminator, and carrying the *Escherichia coli hygB* selectable marker, flanked by the transcriptional control elements of the *trpC* gene from *Aspergillus nidulans*.

**Fig 1:**
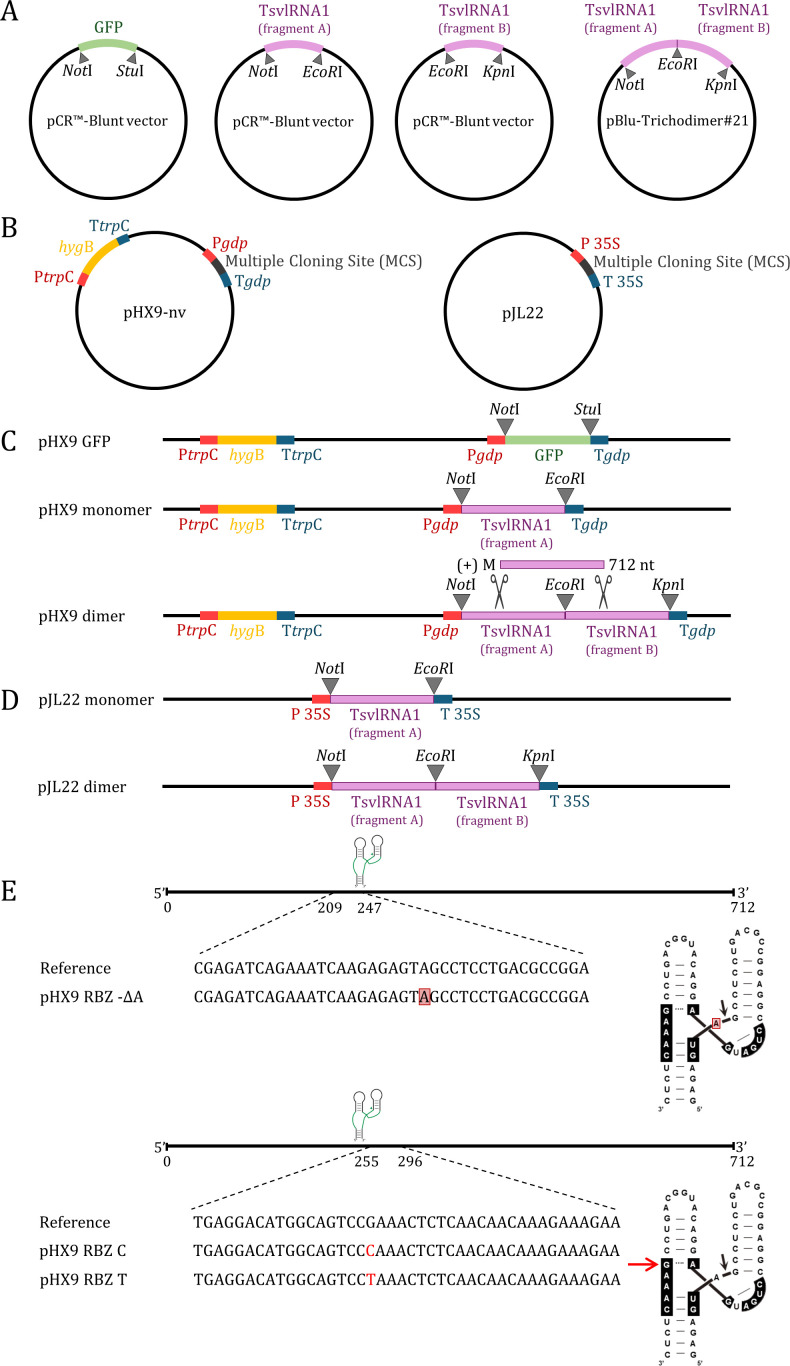
Schematic representation of recombinant plasmids used in this study. The cloning operations used to construct individual expression vectors are described in detail in the Materials and Methods section. Gray triangles indicate restriction sites. (**A**) pCR-Blunt vector containing GFP and TsvlRNA1 fragments A and B, together with the assembled viroid-like RNA dimeric construct (pBlu-Trichodimer#21), used as intermediates to generate the final constructs. (**B**) Backbone plasmids pHX9-nv and pJL22 used for final construct generation. (**C**) Plasmid backbone named pHX9-nv contains *Escherichia coli* hygromycin B phosphotransferase gene (*hygB*) as a selectable marker, flanked by the transcriptional control elements of the *trpC* gene from *Aspergillus nidulans*. GFP gene under the control of *gpd-1* gene promoter and terminator from *C. parasitica* were introduced creating ’pHX9 GFP.‘ Infectious cDNA clones of TsvlRNA1 were generated introducing the monomeric sequence and the dimeric sequence of TsvlRNA1, respectively, flanked by the same promoter and terminator from *C. parasitica*. They are called ‘pHX9 monomer’ and ‘pHX9 dimer.’ The two scissors indicate the position of the hammerhead ribozyme present on the positive strand; its self-cleavage activity generates the (+) monomer, which is 712 nt in length. (**D**) pJL22 plasmid was used to generate two constructs: one containing the monomeric sequence of TsvlRNA1 and the other the dimeric form, named as ‘pJL22 monomer’ and ‘pJL22 dimer.’ Both sequences are flanked by the cauliflower mosaic virus 35S promoter and terminator. (**E**) Recombinant clones derived from “pHX9 dimer” with various mutations targeting the (+) HHrbz active site include a deletion of adenine at position 231 (‘pHX9 RBZ-∆A’) and point mutations substituting cytosine or thymine at position 272 (‘pHX9 RBZ C’ and ‘pHX9 RBZ T,’ respectively). The deletion is indicated by the red box, and the position of the mutations is indicated by the red arrow. In panel E, only one of the two monomers is displayed to allow a more in detail view of the mutations performed in both monomers constituting the dimer clone.

The GFP sequence was amplified from clone R3-p21-TripB-GFP (40) using primers introducing *Not*I and *Stu*I restriction sites, cloned into pCR-Blunt vector (Invitrogen, Carlsbad, CA), and subsequently inserted into *NotI-Stu*I-digested pHX9-nv (Fig. 1).

RNA from *T. spirale* (T45), naturally infected with TsvlRNA1, was reverse transcribed with random primers. Two full-length genomic fragments of TsvlRNA1 (A and B) were PCR-amplified (primers in Table S1) with primers designed to introduce *Not*I*/EcoR*I and *EcoR*I/*Kpn*I restriction sites, respectively, and cloned into two distinct pCR-Blunt vectors (Invitrogen, Carlsbad, CA) (Fig. 1). Fragment A was excised with *Not*I*/EcoR*I and fragment B with *EcoR*I/*Kpn*I, both were used to perform a three-segment ligation with *Not*I*/Kpn*I-digested pBluescript vector using T4 DNA Ligase (Thermo Fisher Scientific, Waltham, MA, USA) to generate plasmid ‘pBlu-Trichodimer#21’ (Fig. 1). The dimer sequence was verified by Sanger sequencing and then excised with *Not*I/*Kpn*I and inserted into pHX9-nv, generating ‘pHX9 dimer’ (Fig. 1).

The pCR-Blunt vector (Invitrogen, Carlsbad, CA) containing fragment A was digested with *Not*I*/Kpn*I (*Kpn*I site from the pCR-Blunt vector), and the excised sequence was inserted into pHX9-nv to generate a transformation vector that expresses a monomer of the vdlRNA molecule (‘pHX9 monomer’) (Fig. 1).

Three TsvlRNA1 mutants—deletion of adenine (A) at position 231 and cytosine (C) or thymine (T) substitutions at position 272—were generated by inverse PCR (Table S1) using both fragments A and B cloned in the pCR-Blunt vector as templates (Fig. 1). The resulting amplicons were digested with the appropriate restriction enzymes and cloned into the pHX9-nv backbone as described for the construction of the ‘pHX9 dimer’ plasmid. This procedure yielded the plasmids ‘pHX9 RBZ -∆A,’ ‘pHX9 RBZ C,’ and ‘pHX9 RBZ T’ (Fig. 1). All the mutated fragment A and B were Sanger sequenced before assembling the dimer.

Each plasmid was used to transform chemically competent DH5α cells, and positive colonies were cultured in liquid Luria-Bertani (LB) medium supplemented with ampicillin (50 µg/mL), and plasmid DNA was subsequently isolated using a ‘ZymoPURE Plasmid Miniprep Kit’ (Zymo Research, Irvine, CA, USA), following the manufacturer’s instructions.

### *Trichoderma* sensitivity test to hygromycin B and protoplasts transformation

The minimum inhibitory concentration (MIC) of hygromycin B required to inhibit *T. lixii*, *T. spirale*, and *T. atrobrunneum* (T36, T45neg, T99) growth was determined on PDA with increasing concentrations and used to select transformants.

Protoplasts derived from T36, T45neg, and T99 were produced and transformed according to Malmierca et al. (41). Transformants were sequentially transferred to medium with twice the MIC of hygromycin B, then to non-selective, and finally to selective medium to obtain mitotically stable isolates, which were grown on cellophane-covered PDA for 4–5 days before mycelia were harvested for RNA extraction.

### GFP visualization

Mycelium from ‘pHX9 GFP’ transformants was transferred onto glass slides. GFP fluorescence was captured with a Zeiss LSM 900 confocal microscope (488-nm excitation, 500–525-nm emission) and analyzed with ZEN 2.3 software (Carl Zeiss AG, Oberkochen, Germany).

### RNA extraction, DNase treatment, cDNA synthesis, and quantitative RT-qPCR

RNA extraction was carried out using the ‘Spectrum Plant Total RNA Kit’ (Sigma-Aldrich, St. Louis, MO, USA) in accordance with the manufacturer’s instructions. RNA concentration was measured using a Nanodrop LITE Spectrophotometer (Thermo Fisher Scientific, Waltham, MA, USA). DNase treatment using ‘DNA-free DNA Removal Kit’ (Ambion, Austin, TX, USA) preceded cDNA synthesis using the ‘High-Capacity cDNA Reverse Transcription Kit’ (Thermo Fisher Scientific, Waltham, MA, USA) with random primers; finally, cDNA was diluted 1:5.

Minus-strand cDNA was generated using a TAG-containing primer (TrichoRBZ TAG NEGS in Table S1) and then purified with the ‘Zymo DNA Clean & Concentrator−25 kit’ (Zymo Research, Irvine, CA, USA).

Primer design was conducted using NCBI primer BLAST tool (https://www.ncbi.nlm.nih.gov/tools/primer-blast/); resulting primers are listed in Table S1.

Minus strand-specific and total cDNA-specific Real-Time qPCR (RT-qPCR) with TsvlRNA1-specific primers/probe were performed on DNase-treated/untreated RNA and corresponding cDNA to verify transgene integration and assess vdlRNA replication. RT-qPCR assays were performed in 10 μL using iTaq Universal Probes Supermix (BioRad, Hercules, USA) on a CFX Connect RT-qPCR Detection System (Biorad, Hercules, USA).

Relative nucleic acid levels were calculated from quantification cycle (Cq) values using the transformation $2^{-Cq}$, and subsequently normalized to the sample with the lowest Cq (highest expression) within each data set. Data are presented as box plots, summarizing the distribution of normalized values for each experimental condition.

### Analyses of RNA self-cleavage *in vitro* and 5′ RACE

The *Not*I- or *Kpn*I-linearized ‘pBlu-Trichodimer#21’ plasmid served as the template for T7 or T3 *in vitro* transcription of TsvlRNA1 dimeric transcripts of plus and minus polarity, respectively. Transcripts were analyzed by denaturing 5% PAGE containing 8 M urea and 1× TBE, and products were recovered for self-cleavage assays and 5′ RACE. TsvlRNA1 primary transcripts were resuspended in Tris-HCl buffer at pH 7.5 or 8.5, denatured at 95°C, gradually cooled to 37°C or 25°C, and then incubated with different MgCl₂ concentrations to induce self-cleavage. For 5′ RACE, the eluted RNA was reverse-transcribed with ‘Superscript IV’ (Invitrogen, Carlsbad, CA) and primer RACEpos349 (Table S1), poly(dG)-tailed, PCR-amplified, cloned, and sequenced according to Hirtzmann methodology (42).

### Northern blot hybridization assays

DNase-treated, denatured RNA was resolved on denaturing 5% PAGE containing 8 M urea and 1× TBE, electroblotted onto nylon membranes, UV-crosslinked, and hybridized with DIG-labeled riboprobes specific for (+) or (−) TsvlRNA1 strands. Riboprobes were synthesized using T7 transcription with the DIG-RNA Labelling Mix (Roche Diagnostics GmbH, Germany) from *SpeI*-linearized pGEM-T Easy containing a partial TsvlRNA1 insert in the correct orientation. Pre-hybridization and hybridization were performed in DIG Easy Hyb (Roche Applied Science, Germany) at 65°C, and signals were detected using anti-DIG alkaline phosphatase antibody fragments and the chemiluminescent substrate CDP-Star (Roche Applied Science, Germany) on a ChemiDoc Touch system (Bio-Rad, Hercules, CA, USA).

### Horizontal and vertical transmission and extracellular acquisition of TsvlRNA1

Donor strains of *T. spirale* (T45DIM5) and *T. atrobrunneum* (T99DIM3) expressing the ‘pXH9 dimer’ were co-cultured with different recipient strains to evaluate whether transgene expression was required for TsvlRNA1 replication (all combinations are listed in Table 1). Donor and recipient strains were placed 40 mm apart on PDA, and recipient plugs collected after 6 and 11 days were subcultured and screened for hygromycin sensitivity. TsvlRNA1 horizontal transmission was then assessed by RT-qPCR and northern blot assays. The same combinations were tested using TsvlRNA1-negative donors (T45neg, T99) to examine interaction dynamics. Vertical transmission was assessed from conidia of TsvlRNA1-positive *T. spirale* (T45ANAS-A) and *T. atrobrunneum* isolates (T84ANAS-1, T99ANAS-1) obtained through co-culture.

**TABLE 1:** Table showing all combinations tested in horizontal transmission experiments between dimer-transformed viroid-like RNA *Trichoderma spirale* (T45DIM5) and *Trichoderma atrobrunneum* (T99DIM3) isolates and viroid-like-free recipient isolates^*a*^

	Recipient							
Donor	T22	T36	T45neg	T71	TO71B	T84	T99	T100
T45DIM5	X	X	✓	✓	X	✓	X	✓
T99DIM3	✓	X	X	✓	✓	✓	✓	X

^*a*^ 'X' indicates absence of TsvlRNA1 839 transmission, while '✓' indicates successful transmission.

To test for possible extracellular acquisition of the viroid-like RNA, T99 mycelia were collected from liquid culture, homogenized in distilled water, washed with STC buffer (1 M sorbitol, 10 mM Tris·HCl, pH 7.5, 20 mM CaCl_2_), and recovered by low-speed centrifugation. Hyphal fragments were mixed with total RNA from T45ANAS-A or *in vitro* produced RNA, incubated on ice and at room temperature for 30 min each, and then grown at 28°C for 4–5 days in liquid medium and recovered for RNA extraction.

### Small RNA sequencing

The small RNAs (sRNAs) of four TsvlRNA1-positive *Trichoderma* isolates—including two also infected by mycoviruses—were sequenced to assess silencing-related antiviral responses (T22ANAS-2, T45ANAS-A, T71ANAS-A, and T99ANAS-2). sRNA libraries were prepared and sequenced on an Illumina NovaSeq X by Novogene Bioinformatic Technology (Beijing, China). The raw sequencing reads were deposited in the NCBI Sequence Read Archive under the BioProject PRJNA1301113 (Accession Number SRR34855465, SRR34855464, SRR34855463, and SRR34855462). After trimming with Trimmomatic (15–50 nt) (43), >10 million reads per sample remained. Reads were mapped to TsvlRNA1 and viral genomes with Burrows-Wheeler Alignment tool (44), allowing circular genome alignment. Sorted BAM (Binary Alignment/Map) files were filtered with SAMtools (45), and strand-specific and size-distribution analyses were generated and visualized with Tablet (46). Figures illustrating small RNA results were generated using the R package viRome (47).

### Evaluation of *Trichoderma* antagonistic activity against fungal infection in small broad bean

Agar plugs bearing mycelium of the soilborne polyphagous pathogen *Rhizoctonia solani* J.G. Kühn (AG-4), previously isolated from quinoa (*Chenopodium quinoa* Willd.), were excised from 7-day PDA cultures and placed onto each *Trichoderma* mycelial plug; both were placed in a seedbed containing sterilized potting mix (Vigorplant Italia). After 1-week, small broad bean (*Vicia faba* var. *minor* Beck) seeds were sown, and the seedbeds were incubated in a glasshouse for 21 days under controlled temperatures and daily irrigation. For each *Trichoderma* isolate, three replicates of nine plants were prepared. After 3 weeks, the disease severity index was assessed using a five-class empirical scale (0–4). A completely randomized design was used, and data were analyzed via one-way ANOVA with Honestly Significant Difference and by Kruskal–Wallis with Dunn’s post-hoc test.

### TsvlRNA1 viroid-like expression in plants

Transient expression of TsvlRNA1 in plants was achieved by cloning its monomeric and dimeric genomic sequences into the binary vector pJL22 (48) under the cauliflower mosaic virus 35S promoter and terminator. Monomer and dimer inserts were excised from ‘pBlu-Trichodimer#21’ using *EcoR*I/*Kpn*I or *Kpn*I/*Not*I, respectively, purified with the ‘Zymoclean Gel DNA Recovery Kit’ (Zymo Research, Irvine, CA, USA), and ligated into linearized pJL22 to generate the constructs ‘pJL22 monomer’ and ‘pJL22 dimer.’ Each construct was used to transform *Agrobacterium tumefaciens* strain C58C1, which was grown on selective medium and resuspended in MES buffer for infiltration.

One-month-old *Nicotiana benthamiana* plants were agroinfiltrated with *A. tumefaciens* carrying ‘pJL22 dimer’ or ‘pJL22 monomer,’ together with clones expressing the silencing suppressor p19 and, in some treatments, the tobacco mosaic virus (TMV) (genus *Tobamovirus*) movement protein. Plants were maintained for 1 week under controlled conditions and monitored for symptoms; infiltrated areas and systemic leaves were collected for RNA extraction using ‘Direct-zol RNA Kits’ (Zymo research, Irvine, CA, USA). Total RNA was DNase-treated, reverse transcribed, and the replication of TsvlRNA1 was detected by minus-strand-specific RT-qPCR.

## RESULTS

### *Trichoderma spirale* harbors an infectious circular viroid-like RNA

**Fig 2:**
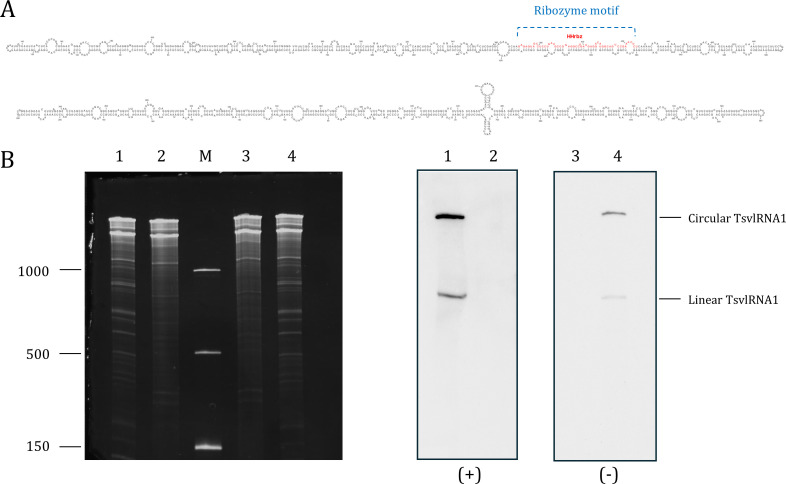
Main molecular features of TsvlRNA1. (**A**) Predicted secondary structure of lowest free energy calculated by RNAfold WebServer (http://rna.tbi.univie.ac.at/cgi-bin/RNAWebSuite/RNAfold.cgi) for the plus and minus strands of Trichoderma spirale viroid-like RNA1. The region involved in the formation of the hammerhead ribozyme structure is delimited by a broken line. (**B**) Detection of circular and linear forms of both polarity strands of TsvlRNA1 by northern blot hybridization assays under denaturing conditions (5% PAGE in 8 M urea 1× TBE). Lanes 1 and 4, RNA preparations from *Trichoderma spirale* isolate T45 tested positive to TsvlRNA1 by HTS and RT-qPCR; lanes 2 and 3 RNA preparations from the TsvlRNA1-negative *Trichoderma* isolate T43. M, RNA ssRNA Ladder (New England Biolabs) with RNA sizes (nt) indicated on the left. Identical aliquots of the same RNA preparations were loaded in parallel in the same PAGE (left, ethidium bromide staining of the gel). After nucleic acid transfer, the membrane was cut vertically and each half was hybridized with equalized DIG-RNA probes specific to detect the plus (lanes 1 and 2) and the minus (lanes 3 and 4) polarity strands of TsvlRNA1 (right end panels). The positions of circular and linear forms of TsvlRNA1 are indicated on the right.

Pagnoni et al. (35) reported the identification of an orphan contig of circa 1.4 kb designated as ORFan2 in *T. spirale* (T45; see Supporting Information and Fig. S1 for details on the identification of all isolates used in this study), which was isolated from an uncultivated soil in Sardinia. Recent bioinformatic analysis indicated that this contig corresponds to a putative circular RNA carrying a HHrbz in one polarity strand (Fig. 2). The monomeric molecule of 712 nucleotides has been renamed Trichoderma spirale viroid-like RNA 1 (TsvlRNA1). *In silico* predictions using RNAfold indicated that the minimum free-energy secondary structure of TsvlRNA1 adopts compact rod-like and quasi rod-like conformation for the plus and minus polarity, respectively (Fig. 2).

**Fig 3:**
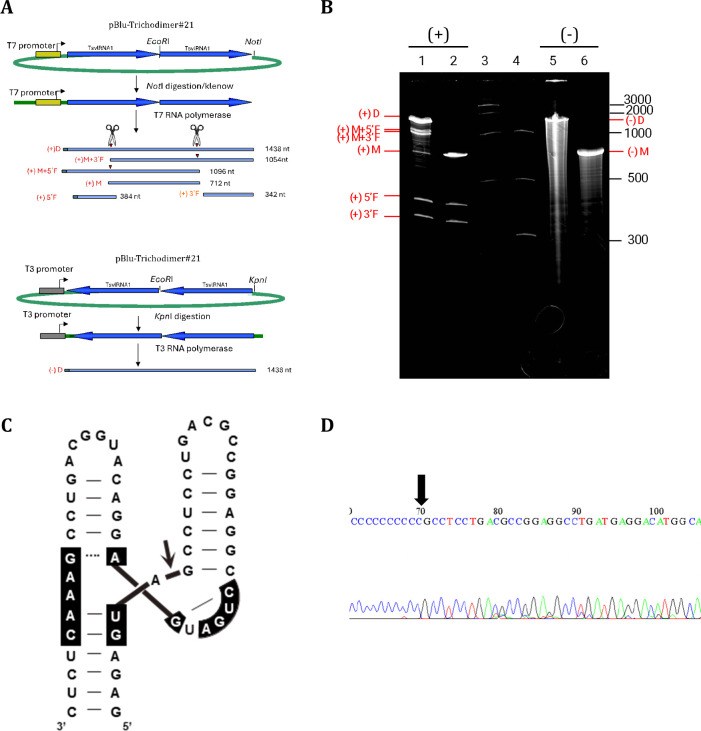
Self-cleavage activity *in vitro* of TsvlRNA1. (**A**) Schematic representation of the plasmid ‘pBlu-Trichodimer#21’, containing the dimeric head-to-tail cDNA sequence of TsvlRNA1, used as template for *in vitro* transcription and the expected RNA products with size reported on the right. Scissors mark the position of the self-cleavage sites. ‘pBlu-Trichodimer#21’ linearized with *EcoR*I and transcribed with T7 or T3 RNA polymerase produces monomeric RNAs (M) of (+) or (−) polarity strands, respectively. (**B**) Analysis by 5% PAGE under denaturing conditions of the *in vitro* transcription of plasmid ‘pBlu-Trichodimer#21’. Lanes 1 and 2, *in vitro* transcription of dimeric and monomeric (+) TsvlRNA1, respectively; lanes 5 and 6, *in vitro* transcription of dimeric and monomeric (−) TsvlRNA1, respectively; Lane 3, ssRNA ladder RNA (New England Biolabs); lane 4, low range ssRNA ladder (New England Biolabs). Sizes in nt are indicated on the right. In red are indicated the RNA fragments generated during transcription (see panel A). (**C**) Secondary structure of (+) hammerhead ribozyme of TsvlRNA1. The arrow indicates the predicted self-cleavage site, and the conserved motifs in most HHrbz are denoted in black background. (**D**) Sanger sequencing electropherogram of the 5′ RACE product of the (+) 3′ fragment generated by (+) TsvlRNA1 HHrbz self-cleavage during *in vitro* transcription. The 5′ terminal nucleotide is indicated by an arrow.

Reverse transcription with random primers followed by PCR using adjacent primers (Table S1) of opposite polarity produced an amplicon of the expected size, supporting the circular nature of TsvlRNA1. Northern blot analysis under denaturing conditions performed on *T. spirale* (T45) RNAs, revealed the presence of monomeric linear and circular forms of both polarity strands *in vivo* (Fig. 2). This finding confirms the circular nature of TsvlRNA1 and supports its replication via a symmetric RCR mechanism, characteristic of members of the *Avsunviroidae* family. This replication variant is associated with two ribozymes, one on each polarity strand. However, TsvlRNA1 contains a known ribozyme solely in the plus strand—by convention, the most abundant *in vivo*. The self-cleaving activity of TsvlRNA1 RNAs of both polarity strands was assessed by *in vitro* transcription of a recombinant plasmid containing head-to-tail dimeric TsvlRNA1 cDNA insert, followed by analysis on denaturing 5% PAGE. During transcription, monomeric and dimeric plus polarity TsvlRNA1 transcripts self-cleaved, generating fragments consistent with (+) HHrbz self-cleavage activity (Fig. 3). Moreover, 5′ RACE experiments followed by cloning and sequencing confirmed that the 5′ termini of the 3′ cleavage fragment matched the predicted ribozyme cleavage site, confirming the specific processing site of the (+) HHrbz (Fig. 3). In contrast, transcripts of the minus polarity strand lacking any identifiable ribozyme did not self-cleave during *in vitro* transcription, showing one band corresponding to the full-length RNA transcript (Fig. 3). Attempts to induce post-transcriptional *in vitro* self-cleavage of purified dimeric (-) TsvlRNA1 transcripts by denaturation and slow renaturation under high Mg²^+^ and varied pH conditions were unsuccessful. These findings suggest either the existence of a yet undiscovered ribozyme with specific activation requirements or the involvement of a host fungal RNase in the replication of this vdlRNA.

Within the TsvlRNA1 sequence, four putative Open Reading Frames (ORFs) were identified (Fig. S2). Two of these, designated ORF1 and ORF2, are located on the plus strand and are predicted to encode peptides of approximately 9.64 kDa (84 amino acids) and 3.25 kDa (30 amino acids), respectively. The other two, ORF3 and ORF4, are located on the minus strand and are predicted to encode peptides of approximately 9.91 kDa (87 amino acids) and 6.62 kDa (60 amino acids), respectively. None of these putative ORFs exhibit significant similarity to any known proteins in current databases.

### TsvlRNA1 replication is initiated from the *in vivo* transcription of dimer viroid-like cDNA

**Fig 4:**
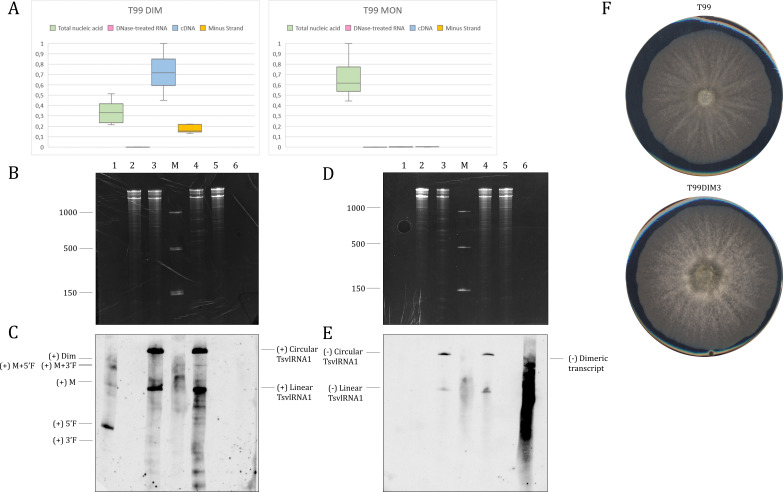
Detection of TsvlRNA1 by Real-Time qPCR and northern blot hybridization in transgenic fungal isolates. (**A**) Box plots showing the distribution of normalized relative quantification cycle (Cq) obtained from Real-Time qPCR analysis. Each category includes data from three independent transformants, each analyzed with two technical replicates of the Real-Time qPCR. In all panels, the ‘Total nucleic acid’ category indicates successful integration of the transgene across samples. The ‘DNase-treated RNA’ values confirm the effectiveness of DNase treatment. The ‘cDNA’ and ‘Minus strand’ categories reflect the replication capacity of each infectious clone within each isolate. The panel shows the isolate T99 transformed with ‘pHX9 dimer’ (T99 DIM) and ‘pHX9 monomer’ (T99 MON). (**B–E**) Northern blot assays were performed under denaturing conditions (5% PAGE in 8 M urea 1× TBE). Lane 1 *in vitro* transcription of dimeric (+) TsvlRNA1, lane 2 RNA preparations from the untransformed and TsvlRNA1-negative *Trichoderma atrobrunneum* isolate T99, lane 3 RNA preparations from *T. spirale* isolate T45 which tested positive to TsvlRNA1 by HTS and RT-qPCR, lane 4 RNA preparations from *T. atrobrunneum* isolate T99 transformed with ‘pHX9 dimer,’ lane 5 RNA preparations from *T. atrobrunneum* isolate T99 transformed with ‘pHX9 monomer,’ and lane 6 *in vitro* transcription of dimeric (−) TsvlRNA1. M, low range ssRNA Ladder (New England Biolabs) with RNA sizes (nt) indicated on the left. Identical aliquots of the same RNA preparations were loaded in the PAGEs shown in panels (**B**) and (**D**). (**B–D**) Ethidium bromide staining of the gel. After nucleic acid transfer, the membrane was hybridized with equalized DIG-RNA probes specific to detect the plus (panel **C**) and the minus (panel **E**) polarity strands of TsvlRNA1. The positions of circular and linear forms of TsvlRNA1 are indicated. (**F**) Top views of PDA plates of TsvlRNA1-free *T. atrobrunneum* (T99) and TsvlRNA1-positive transformed *T. atrobrunneum* (T99DIM3) after 48 h post-incubation at 28°C.

We initially engineered a monomer and head-to-tail dimer cDNA of TsvlRNA1 in the fungal expression vector pXH9. Plasmids ‘pHX9 dimer’ and ‘pHX9 monomer’ were generated incorporating into pHX9-nv backbone the vldRNA dimeric and monomeric sequence, respectively (Fig. 1). Transformants from isolates *T. lixii* (T36), *T. spirale* (T45neg), and *T. atrobrunneum* (T99) showing mitotic stability for hygromycin B resistance were analyzed to confirm transgene integration through qPCR using TsvlRNA1-specific primers (Table S1) on total nucleic acid samples (Fig. 4 and Table S3).

Northern blot analysis using probes for both plus and minus polarities was performed on DNase-treated RNA samples from the transformants. The analysis revealed the presence of monomeric linear and circular forms of both polarities in the ‘pXH9 dimer’ but not in the ‘pXH9 monomer’ transformants (Fig. 4). These results provide evidence of replication of TsvlRNA1 and support that its replication is initiated only in the presence of the dimeric transgene generating the genomic monomeric linear forms by self-cleavage (Fig. 1). Some differences in colony morphology were observed, and TsvlRNA1-infected T99 isolates generated via transformation (T99DIM3, T99DIM25, and T99DIM52) exhibited a slightly higher radial growth rate on PDA plates after 72 h of incubation at 28°C compared with the viroid-free T99 isolate (Fig. 4 and Fig. S3).

**Fig 5:**
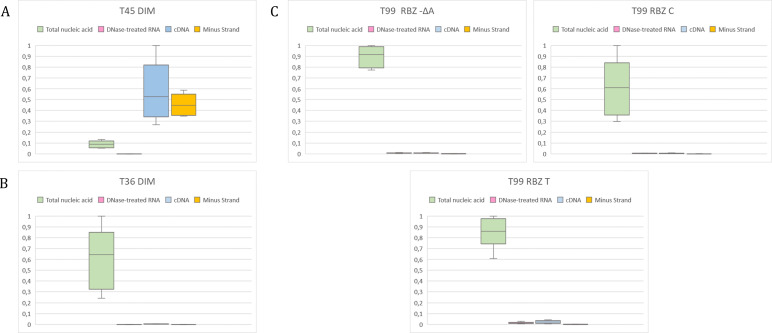
Quantitative analysis of transgene expression and viroid replication based on normalized Real-Time qPCR data. Box plots showing the distribution of normalized relative quantification cycle (Cq) values obtained from Real-Time qPCR analysis. Each category includes data from three independent transformants, each analyzed with two technical replicates of the Real-Time qPCR. In all panels, the ‘Total nucleic acid’ category indicates successful integration of the transgene across samples. The ‘DNase-treated RNA’ values confirm the effectiveness of DNase treatment. The ‘cDNA’ and ‘Minus strand’ categories reflect the replication capacity of each infectious clone within each isolate. Panel (**A**) shows the isolate T45 transformed with ‘pHX9 dimer’ (T45 DIM). Panel (**B**) shows the isolate T36 transformed with ‘pHX9 dimer’ (T36 DIM). Panel (**C**) shows the isolate T99 transformed with three plasmids carrying mutations in the catalytic core of the ribozyme (T99 RBZ -ΔA, T99 RBZ C, and T99 RBZ T).

However, the infectivity of the dimeric construct does not apply to *T. lixii* (T36); although the transformation was successful, minus strand-specific RT-qPCR negative results revealed the absence of TsvlRNA1 replication (Fig. 5 and Table S3).

### Infectious clone mutants of TsvlRNA1

To evaluate the functional relevance of the (+) HHrbz of TsvlRNA1 for replication, targeted mutagenesis was employed to affect ribozyme self-cleavage. Plasmids carrying a dimeric TsvlRNA1 insert with a nucleotide deletion or two distinct single point mutations in the HHrbz catalytic core were used for transformation (‘pHX9 RBZ -∆A,’ ‘pHX9 RBZ C,’ and ‘pHX9 RBZ T’) (Fig. 1).

Protoplasts from *T. atrobrunneum* (T99) were transformed with all three plasmids. Although transgene integration was confirmed by qPCR, no TsvlRNA1 replication was detected by minus strand-specific RT-qPCR (Fig. 5 and Table S3). Results obtained with the infectious clone mutants of TsvlRNA1 confirm that a catalytically active core within the ribozyme is essential for TsvlRNA1 replication, as mutations in this region are predicted to prevent cleavage function and consequently the formation of the linear form during the RCR mechanism (49).

### Horizontal transmission of TsvlRNA1

A critical test for establishing vdlRNAs infectivity is their capacity to disseminate throughout the host population. In general, horizontal transmission of mycoviruses occurs through anastomosis (hyphal fusion). We, therefore, conducted experiments using dimer-transformed viroid-like isolates and viroid-like-free recipient isolates (all combinations are shown in Table 1).

**Fig 6:**
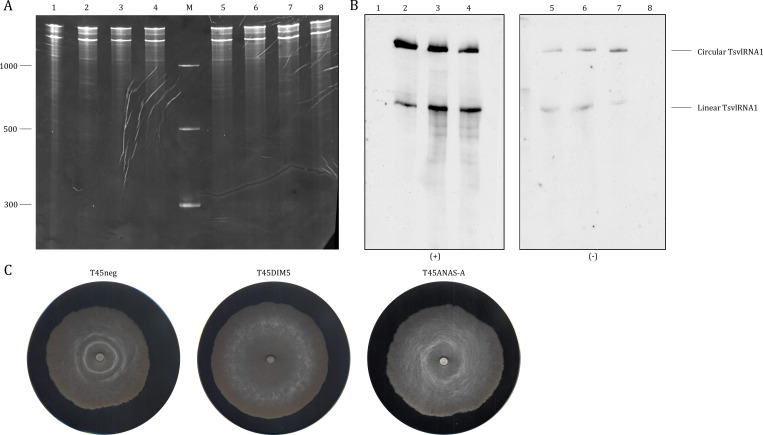
Detection of circular and linear forms of both polarity strands of TsvlRNA1 in horizontally transfected isolates by northern blot. Hybridization assays were carried out under denaturing conditions (5% PAGE in 8 M urea 1× TBE). Lanes 1 and 8 RNA preparations from the TsvlRNA1-negative *Trichoderma spirale* isolate T45neg, lanes 2 and 7 RNA preparations from *T. spirale* isolate T45 which tested positive to TsvlRNA1 by HTS and RT-qPCR, lanes 3 and 6 RNA preparations from *T. spirale* isolate T45DIM5 transformed with ‘pHX9 dimer,’ lanes 4 and 5 RNA preparations from *T. spirale* isolate T45ANAS-A obtained through horizontal transmission. M, RNA ssRNA Ladder (New England Biolabs) with RNA sizes (nt) indicated on the left. Identical aliquots of the same RNA preparations were loaded in parallel in the same PAGE. (**A**) Ethidium bromide staining of the gel. After nucleic acid transfer, the membrane was cut vertically and each half was hybridized with equalized DIG-RNA probes specific to detect the plus (lanes 1, 2, 3, and 4) and the minus (lanes 5, 6, 7, and 8) polarity strands of TsvlRNA1 (Panel **B**). The positions of circular and linear forms of TsvlRNA1 are indicated on the right. (**C**) Top views of PDA plates of TsvlRNA1-free *T. spirale* (T45neg), TsvlRNA1-positive transformed *T. spirale* (T45DIM5) and TsvlRNA1-positive obtained through co-culture *T. spirale* (T45ANAS-A) after 72 h post-incubation at 28°C.

The presence of TsvlRNA1 in recipient strains was confirmed through RT-qPCR and minus strand-specific RT-qPCR using as template cDNA synthesized from RNA extracted from colonies unable to grow on hygromycin B-containing medium (Table S4). When *T. atrobrunneum* (T99DIM3) transformed with the plasmid ‘pXH9 dimer’ was the donor strain, TsvlRNA1 was transmitted to *T. afroharzianum* (T22, TO71B), *T. gamsii* (T71), *T. atrobrunneum* (T84, T99); when *T. spirale* transformed with the same plasmid (T45DIM5) was the donor, transmission occurred to *T. spirale* (T45neg), *T. gamsii* (T71), *T. atrobrunneum* (T84), and *T. velutinum* (T100). Transmission failures were observed between certain donor-recipient pairs (Table 1). Northern blot analysis confirmed monomeric linear and circular forms of both strands in recipient strains, demonstrating that TsvlRNA1 is a replicative and infectious RNA (Fig. 6). As observed for *T. atrobrunneum*, *T. spirale* transformed with the dimeric construct (T45DIM5) exhibited the highest growth rate on PDA plates at 28°C after 48 h compared with the vdlRNA-free isolate (T45neg), whereas the TsvlRNA1-positive isolate obtained through co-culture (T45ANAS-A) showed an intermediate growth profile, with no statistically significant difference compared to the other two isolates (Fig. 6 and Fig. S4).

These results show that TsvlRNA1 can spread horizontally in *Trichoderma* species, resulting in isogenic, transgene-free infected colonies (Fig. S5 shows the absence of transgene amplification), and provide the first evidence of transmission to species beyond its original host, *T. spirale*. None of the fungal species’ pairings tested for TsvlRNA1 transmission showed evidence of barrage (incompatible reactions), supporting the possibility that interspecific TsvlRNA1 transmission relies on anastomosis of compatible hyphal fusion among different species (see Fig. S6 and Supporting Information for details).

Based on prior virome characterization (35), some of the isolates we are using harbor mycoviruses (Table S5). Nevertheless, successful horizontal transmission of TsvlRNA1 from the virus-free donor strain of *T. spirale* (T45DIM5) to the virus-free recipient strains *T. spirale* (T45neg), *T. gamsii* (T71), and *T. velutinum* (T100) indicates that, like viroids, TsvlRNA1 replication does not rely on a helper virus. Although T84 and T99 are both classified as *T. atrobrunneum*, TsvlRNA1 transmission from T45DIM5 was detected only in T84, suggesting that strain-level differences, may affect TsvlRNA1 transmissibility (Table S4). When T99DIM3 was used as the donor, the present mycoviruses were co-transmitted together with the vdlRNA to different *Trichoderma* species (Table S5).

### Assays for vdlRNA vertical transmission and acquisition of environmental RNA

Vertical transmission of TsvlRNA1 was assessed in conidial progeny of infected isolates. Single-conidium-derived colonies from TsvlRNA1-positive *T. spirale* (T45ANAS-A) and *T. atrobrunneum* isolates (T84ANAS-1 and T99ANAS-1), obtained via horizontal transfer, were analyzed. RT-qPCR analysis revealed that none of the progeny derived from T84ANAS-1 and T99ANAS-1 carried TsvlRNA1, whereas approximately 50% of the progeny from T45ANAS-A tested positive, although they generally exhibited a lower titer (Table S6).

All colonies derived from T99 hyphal fragments exposed to total RNA from TsvlRNA1-positive T45ANAS-A or to vdlRNA transcripts synthetized *in vitro* tested negative in northern blot analysis. These results indicate that TsvlRNA1 is vertically transmitted via conidia only in its original host (*T. spirale*) and cannot be acquired from environmental RNA.

### RNAi response to TsvlRNA1

**Fig 7:**
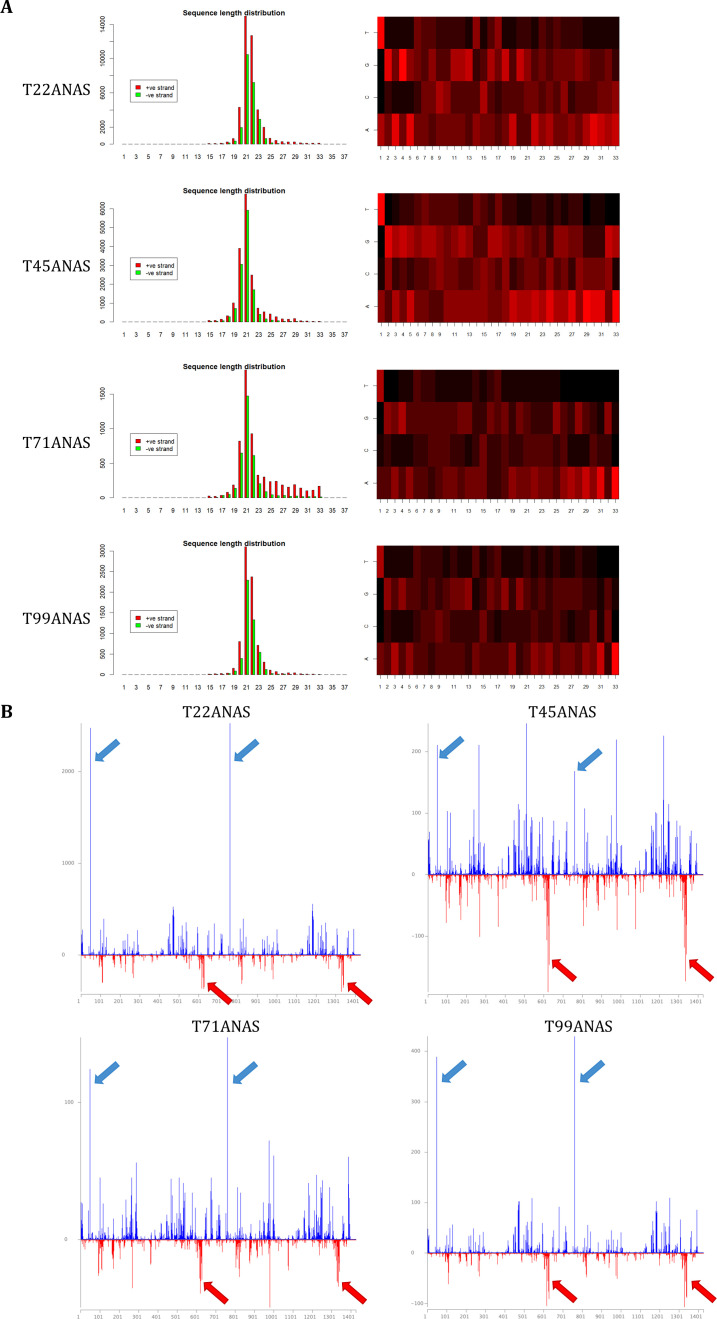
Small RNAs analysis revealed a canonical anti-viral silencing response. (**A**) On the left, size distribution of TsvlRNA1-derived plus (red) and minus sense (green) small RNAs in isolates T22ANAS, T45ANAS, T71ANAS, and T99ANAS. In all cases, plus-strand sRNAs accounted for a slightly higher proportion than minus-strand sRNA and a predominant 21-nt peak is observed. On the right, heatmap representations illustrating nucleotide frequency at each position along the small RNA length. (**B**) Genomic distribution of TsvlRNA1-derived small RNAs mapped along the dimeric TsvlRNA1 sequence in isolates T22ANAS, T45ANAS, T71ANAS, and T99ANAS. The profile highlights the spatial distribution of sRNA coverage across the viroid-like RNA genome, revealing two main hotspots: within nucleotides 47–67 on the plus strand and between nucleotides 620–647 on the minus strand, with minor variation among isolates.

Small RNAs profiles from TsvlRNA1 were compared with those of co-infecting mycoviruses across multiple *Trichoderma* isolates, including two virus-free isolates (T45ANAS-A and T71ANAS-A) and two isolates harboring cytoplasmic mycoviruses (T22ANAS-2 and T99ANAS-2): specifically they both harbor Trichoderma harzianum negative-stranded virus 1 (ThNV1), Trichoderma gamsii alphapartitivirus 1 (TgAPV1), and Trichoderma gamsii mycobunyavirus 1 (TgMBV1) (Table S5). Viroid-like-derived small RNAs (vd-sRNAs) were mainly 20–22 nt long, with 21-nt species predominating across all isolates, whereas the second most prominent peak corresponded to 22 nt in all isolates except T45ANAS-A, in which a 20-nt peak prevailed (Fig. 7). Plus-strand sRNAs were slightly more abundant than minus-strand sRNAs, consistent with the preferential accumulation of plus polarity RNA species in the host (Fig. 7). Heatmap analysis representing nucleotide frequency at each position along the sRNA length revealed that thymine (T) was the most frequent initiating nucleotide at the 5′ terminus, followed by adenine (A) as the second most common (Fig. 7). Mapping of TsvlRNA1-derived sRNA reads identified several hotspots conserved across different species and without clear functional association: between nucleotides 47 and 67 on the plus strand, and between nucleotides 620 and 647 on the minus strand (Fig. 7). Collectively, small interfering RNA analysis demonstrates the activation of the RNAi-mediated antiviral mechanism against TsvlRNA1 infection.

A comparable pattern was observed for virus-derived sRNAs in isolates T22ANAS-2 and T99ANAS-2, which closely mirrored the distribution described above, with a dominant 21-nt peak (Fig. S7). Notably, these virus-derived sRNAs also exhibited a predominant thymine (T) at the first 5′ nucleotide (Fig. S7), suggesting that the same fungal RNA silencing machinery is likely responsible for processing both viral and viroid-like-derived sRNAs. An exception was observed for ThNV1-derived sRNA, which displayed a less abundant sRNA accumulation and less defined size distribution (Fig. S7). This atypical profile may reflect a differential activity of a viral suppressor of RNA silencing encoded by ThNV1, potentially interfering with the canonical Dicer-mediated processing pathway, or uncanonical targeting of this virus through alternative sRNA generating mechanisms.

### Effect of TsvlRNA1 infection on antagonistic capacity of *Trichoderma* species

**Fig 8:**
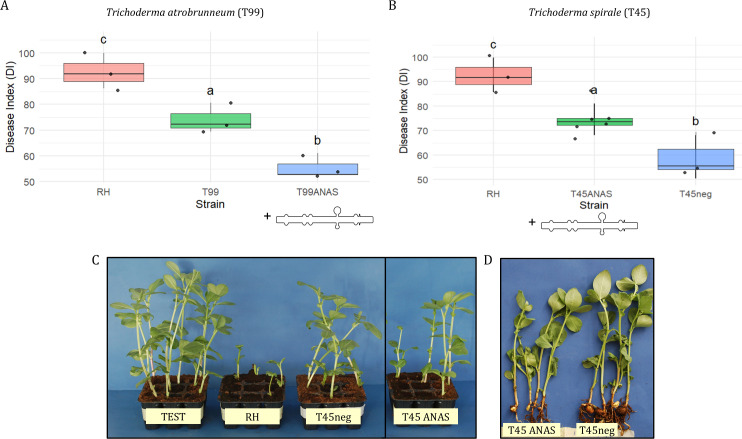
Box plots illustrate the assessment of *Trichoderma* antagonistic activity against *Rhizoctonia solani* infection in small broad beans. In the box plots, the data points represent aggregated values derived from three biological replicates, each consisting of nine plants; results from experimental replicates corresponding to isolates obtained by co-culture (T45ANAS-A and -C, T99ANAS-1) were combined prior to analysis. For *R. solani* control (RH) and the original recipient isolates (T45neg and T99), the data derive from three biological replicates. Statistical analyses were performed using one-way ANOVA followed by Honestly Significant Difference post-hoc test and the Kruskal–Wallis test followed by Dunn’s post-hoc test. Strains infected by the TsvlRNA1 are represented by a graphical symbol under the name of the isolate in the *X* axis. (**A**) A positive effect of TsvlRNA1 presence on the ability of *T. atrobrunneum* (T99ANAS-1) to limit the *R. solani* caused symptoms compared to the same strain without TsvlRNA1 (T99). (**B**) Reduction of the antagonistic capacity for strains of *T. spirale* with TsvlRNA1 presence (T45ANAS-A and -C) to limit the *R. solani* symptoms. DI: Disease index. Strain sharing the same letters are not statistically different. Panel (**C**) shows the nine plants from a biological replicate for the TEST (untreated plants), RH (plants inoculated only with *R. solani*), T45 neg, and T45ANAS treatments at the end of the antagonism assay. Panel (**D**) depicts the roots and collar region of plants treated with T45 neg and T45ANAS.

Since *Trichoderma* genus is commonly used as a biocontrol agent in agriculture, different pairs of TsvlRNA1-infected and TsvlRNA1-free isogenic *Trichoderma* strains obtained from co-culture (T45ANAS-A and -C, T71ANAS-2, TO71BANAS-1 and -2, T84ANAS-1 and -A, and T99ANAS-1) were evaluated for their capability to reduce the damage caused by *R. solani* on small broad bean seedlings. Although several strains harbored multiple mycoviruses (Table S5), complicating attribution of biological effects exclusively to the vdlRNA element, results consistently suggested that TsvlRNA1 infection modulates antagonistic activity (Fig. S8). Among the cases in which the specific contribution of the vdlRNA could be more clearly discerned, TsvlRNA1 significantly enhanced antagonism in *T. atrobrunneum* (T99ANAS-1) against pre- and post-emergence damping-off (Fig. 8A). Indeed, the T99 isolate reduced disease by approximately 20%, while the TsvlRNA1-infected T99ANAS-1 treatment achieved a stronger suppression of about 40%–45% compared to plants inoculated only with *R. solani*. Instead, in *T. spirale,* the effect on antagonistic activity was negative (Fig. 8B): T45ANAS-A and -C reduced disease severity by roughly 20%, whereas the TsvlRNA1-free T45neg condition resulted in a greater reduction of approximately 35%–40%.

### No evidence of TsvlRNA1 replication in plant cell

The same monomeric and dimeric TsvlRNA1-cDNA molecules used for *Trichoderma* experiments were cloned between the 35S promoter and terminator of the binary vector pJL22 for expression in *N. benthamiana* via *A. tumefaciens*. Seven days post agroinfiltration, no significant differences in plus-strand accumulation were detected between the constructs, and minus strand accumulation was undetectable for both constructs in infiltrated areas and systemic leaves (Table S7). Local complementation with TMV movement protein did not affect RNA accumulation.

## DISCUSSION

This study reports the first *in silico* identification of a vdlRNA, called TsvlRNA1, in the non-phytopathogenic fungus *T. spirale*. Diener’s proposal that viroids might represent relics of the primordial RNA world was regarded with skepticism, as confirmed hosts were restricted to plants and later to animals (50, 51). Recent metatranscriptomic studies, including this work, have reshaped this perspective by identifying fungal vdlRNAs, comprising ambiviruses and mitoviruses (5, 52, 53), and Obelisks in bacteria (22). Nevertheless, assessing common ancestry of these agents remains challenging due to their low level of sequence conservation (53).

The circular nature of TsvlRNA1 is supported by PCR amplification of a full-length product with adjacent primers of opposite polarity, northern blot detection of circular monomers of both polarities *in vivo*, and bioinformatic identification of direct repeated sequences. Although the detection of circular and linear RNA monomers of both polarities is indicative of a symmetric RCR mechanism involving ribozymes in both polarity strands (2), only a single HHrbz in the plus strand of TsvlRNA1 was predicted and experimentally validated *in vitro*, while no ribozyme or cleavage activity was detected in the minus strand. This represents the first example of a vdlRNA undergoing symmetric RCR while producing circular RNAs of both polarities despite having a ribozyme in only one strand. Although the vdlRNAs described by Dong et al. (52) from *Botryosphaeria dothidea* accumulate circular and linear forms of both strands, consistent with our observations, they lack recognizable ribozymes, and only one of them exhibits *in vitro* self-cleavage of one strand. This finding deviates from the canonical symmetric RCR model and suggests a replication strategy partially resembling that of certain viroid-like satellite RNAs (e.g., *Solanum nodiflorum* mottle virus and subterranean clover mottle virus) (15), or likely involving an as-yet unidentified ribozyme or a fungal host-derived RNase. This mechanistic discrepancy remains unresolved and warrants further investigation.

To establish a reverse genetic system, infectious cDNA clones of TsvlRNA1 were generated, representing, to our knowledge, the first fungal vdlRNA clone with an experimentally validated ribozyme. Head-to-tail dimeric construct was essential for replication, whereas monomeric sequence was non-infectious, consistent with plant viroid studies (54). Replication was confirmed via RT-qPCR and northern blot analysis, and horizontal transmission to cDNA-free strains demonstrated autonomous replication independent of the transgene and/or helper virus. Mutant infectious clones affecting TsvlRNA1 HHrbz catalytic activity demonstrated that a functional ribozyme is essential for replication.

TsvlRNA1 can horizontally transmit and replicate autonomously across different *Trichoderma* species, independent of co-infecting mycoviruses, as demonstrated by transferring from a virus-free donor to three different virus-free recipient species. These results indicate that TsvlRNA1 is not a viroid-like satellite RNA, but rather an infectious agent endowed with autonomous replication within the infected cells. Considering that mycoviruses are typically transmitted via hyphal fusion between isolates of the same VCG and that intraspecies transmission is restricted by VIC (30), the observed horizontal transmission of TsvlRNA1 and other viruses among different *Trichoderma* species belonging to distinct sections—taxonomic units comprising evolutionarily related species separated by substantial genetic and temporal distances—is noteworthy (55). Moreover, to the best of our knowledge, no studies have specifically addressed interspecific interactions among the species examined here. It is plausible that previous studies have overestimated the ability of VIC to inhibit virus transmission, as on-site investigations indicate that mycoviruses can spread efficiently among vegetatively incompatible strains, suggesting that environmental factors may facilitate transmission (56). Despite evidence that extracellular RNAs can persist in a stable and biologically active form (57, 58), our results indicate that environmental acquisition of TsvlRNA1 does not represent a mode of transmission. Therefore, additional transmission mechanisms, such as the secretion and uptake of extracellular vesicles, cannot be excluded. As vertical transmission was detected only in the original host (*T. spirale*), horizontal transmission appears to represent the predominant mode of dissemination for TsvlRNA1, plausibly enabling its long-term persistence within natural fungal populations.

Among the *Trichoderma* isolates tested, *T. lixii* (T36) was the only strain in which TsvlRNA1 replication was undetectable despite successful integration of the dimeric transgene. Furthermore, vdlRNA transmission to T36 did not occur from either *T. spirale* (T45DIM5) or *T. atrobrunneum* (T99DIM3). This resistance may reflect differences in host factors required for TsvlRNA1 replication.

RNA silencing represents a eukaryotic defense against invasive nucleic acids, triggered by double-stranded RNAs (dsRNAs) or structured single-stranded RNAs (ssRNAs) and mediated by Dicer-like ribonucleases (59). Viroids are strong inducers of this response due to their circular, highly base-paired ssRNA genome and replication via dsRNA intermediates (60). We provide the first analysis of sRNA populations in fungi infected by a vdlRNA. All TsvlRNA1-positive isolates accumulated 20–22 nt sRNAs with a dominant 21-nt peak, regardless of co-infection with other mycoviruses. This profile resembles sRNA patterns observed during infection by cytoplasmically replicating mycoviruses (61) and chloroplast-replicating viroids of the *Avsunviroidae* family (62), which are characterized by the accumulation of 19–22 nt sRNAs, peaking at 21 nt, and of 21–22 nt sRNAs, respectively. This reflects a conserved RNA silencing response across diverse eukaryotic lineages.

*Trichoderma* spp. are widely used biocontrol agents (26) and the investigation of their antagonistic capacity against *R. solani*, a destructive necrotrophic plant pathogen, is of major interest. Antagonistic trials using isogenic *Trichoderma* strains revealed that TsvlRNA1 can modulate biocontrol efficacy either positively or negatively in a strain/species-dependent manner; moreover, in most tested strains, co-infecting mycoviruses hindered the specific effect of TsvlRNA1. Enhanced antagonism in *T. atrobrunneum* (T99ANAS-1) suggests potential applications in fungal bioformulations, whereas the negative effect in *T. spirale* (T45ANAS-A and -C) highlights risks associated with field deployment, particularly due to possible horizontal transmission to native species and unpredictable outcomes.

The proven phenotypic effect of the vdlRNA alone, or in interactions with mycoviruses, suggests that searching for such elements through well-established bioinformatic pipelines should be a gold standard in studies that associate genotype to phenotype. These elements are functional components of fungal microbiomes and can be lost or gained experimentally when evaluating other fungal phenotypic features (site directed mutagenesis, mycovirus transfection/curing, etc.). Our study implies that ribozyme detection in a single orientation should be included in vdlRNA detection bioinformatic pipelines, increasing the number of vdlRNAs previously overlooked.

Our findings on horizontal transmission among different *Trichoderma* species are somewhat surprising since they encompass both vdlRNA and mycoviruses. Only occasional indirect evidence has been reported in the literature regarding the isolation of the same viral species from different fungal hosts, in one case belonging to distinct classes (63–66). Moreover, interspecific transmission in *in vitro* co-culture experiments has also been sporadically documented (65, 67, 68). To further support our findings from controlled-environment experiments, we noticed that in the *Trichoderma*-associated virome data set reported by Pagnoni et al. (35), several mycoviruses were detected in different *Trichoderma* species originating from the same geographical region (Table S8). Given the importance of horizontal transmission of cytoplasmic elements for resistance to antifungals (a sanitary emergency world-wide) (69, 70), we propose to establish a complex interspecific *Trichoderma* hyphal network monitoring mycovirus and vdlRNA transmission as an experimental system to study the mechanism of interspecific hyphal fusion. It is worth speculating that the mycoparasitic lifestyle of *Trichoderma* spp. may confer a specific propensity for hyphal fusion, potentially bypassing the pre- and post-fusion checkpoints characterized in other genera within the Pezizomycotina subphylum (71, 72).

## Data Availability

The resulting raw sequencing reads have been deposited in the NCBI Sequence Read Archive (SRA) under BioProject PRJNA1303385 (Accession Number SRR34918256), and PRJNA1301113 (Accession Number SRR34855465, SRR34855464, SRR34855463, and SRR34855462).

## SUPPLEMENTAL MATERIAL

Supplemental material mbio.00221-26-s0001.docx Supplemental text, figures, and tables.

ASM does not own the copyrights to Supplemental Material that may be linked to, or accessed through, an article. The authors have granted ASM a non-exclusive, world-wide license to publish the Supplemental Material files. Please contact the corresponding author directly for reuse.
